# Triage and action minor emergency: A simulation course to learn the initial treatment of minor emergencies

**DOI:** 10.1002/jgf2.361

**Published:** 2020-07-18

**Authors:** Tomoyasu Matsubara, Kenji Numata

**Affiliations:** ^1^ Department of Clinical Neuroscience and Therapeutics Hiroshima University Graduate School of Biomedical and Health Sciences Hiroshima Japan; ^2^ Department of Emergency Medicine Tokyo Bay Urayasu Ichikawa Medical Center Chiba Japan

To the Editor,

Comprehensive care is central to a primary care doctor’s practice,^1^ and primary care physicians should strive to listen to and manage a wider range of complaints and problems. In this respect, treatment of some of the minor emergencies, including some common surgical procedures, is a skill set that every primary care physician should acquire. However, until now, there had been insufficient training programs in Japan for primary care doctors to comprehensively study the treatment of minor emergencies. Therefore, we developed the “Triage and Action (T&A) Minor Emergency Course,” a simulation course to manage the initial treatment of minor emergencies, in 2015.^2^

The course consists of lectures, demonstrations, and simulation‐based training. The diseases discussed in this course were selected based on urgent but common problems encountered in primary care settings^3^; emergency conditions such as ocular surface foreign bodies, central retinal artery occlusion, epistaxis, foreign bodies in the ear, minor burns, animal bites, sprains, and fractures are included. This is a one‐day , six‐hour  course that includes lectures on eight items and simulation‐based training on five of them. Two T&A Minor Emergency Course instructors typically supervise five participants.

The teaching methods used also employ several features to facilitate learning. First, this course is intended for learners with little experience treating minor emergencies, and it uses role‐playing so that the participants can learn the treatment flow as well as the surgical procedures. Second, we have instructors from a diverse set of specialties: not only family physicians and emergency physicians but also specialists from ophthalmology, otolaryngology, dermatology, and orthopedics. Their involvement provides a greater advantage in explaining the details of procedures and the follow‐up treatments performed by the specialists. In addition, sufficient time (one hour) is allocated for a question‐and‐answer session to facilitate active discussions between the experts and the participants. We believe this approach helps address any uncertainties during the course and allows for more in‐depth discussions.

The course has been conducted 33 times since 2015, and 940 doctors had completed the course as of March 31, 2020. We have received extremely positive feedback from the participants, with 98.4% (303 out of 308 participants) rating it as “Excellent” or “Good” on a 5‐point Likert scale postcourse questionnaire over the last 10 sessions held between April 2019 and January 2020.

In summary, we have developed a “T&A Minor Emergency Course” to help primary care physicians learn the basics of minor emergency treatments. We hope that using this curriculum, primary care doctors will be able to master the basics of minor surgical treatment more efficiently and steadily.

## CONFLICT OF INTEREST

The authors have stated explicitly that there are no conflicts of interest in connection with this article.
